# Mechanistic Insights into the Antibiofilm Mode of Action of Ellagic Acid

**DOI:** 10.3390/pharmaceutics15061757

**Published:** 2023-06-17

**Authors:** Alessandro Ratti, Enrico M. A. Fassi, Fabio Forlani, Matteo Mori, Federica Villa, Francesca Cappitelli, Jacopo Sgrignani, Gabriella Roda, Andrea Cavalli, Stefania Villa, Giovanni Grazioso

**Affiliations:** 1 Dipartimento di Scienze Farmaceutiche, Università degli Studi di Milano, Via L. Mangiagalli 25, 20133 Milano, Italy; alessandro.ratti@unimi.it (A.R.); enrico.fassi@unimi.it (E.M.A.F.); matteo.mori@unimi.it (M.M.); gabriella.roda@unimi.it (G.R.); stefania.villa@unimi.it (S.V.); 2 Dipartimento di Scienze per gli Alimenti, la Nutrizione e l’Ambiente, Via G. Celoria 2, 20133 Milano, Italy; federica.villa@unimi.it (F.V.); francesca.cappitelli@unimi.it (F.C.); 3 Institute for Research in Biomedicine (IRB), Università della Svizzera Italiana (USI), Via Chiesa 5, 6500 Bellinzona, Switzerland; jacopo.sgrignani@irb.usi.ch (J.S.); andera.cavalli@irb.usi.ch (A.C.); 4 Swiss Institute of Bioinformatics, 1015 Lausanne, Switzerland

**Keywords:** ellagic acid, antibiofilm, WrbA, molecular dynamics, polyphenols, *E. coli*

## Abstract

Bacterial biofilm is a major contributor to the persistence of infection and the limited efficacy of antibiotics. Antibiofilm molecules that interfere with the biofilm lifestyle offer a valuable tool in fighting bacterial pathogens. Ellagic acid (EA) is a natural polyphenol that has shown attractive antibiofilm properties. However, its precise antibiofilm mode of action remains unknown. Experimental evidence links the NADH:quinone oxidoreductase enzyme WrbA to biofilm formation, stress response, and pathogen virulence. Moreover, WrbA has demonstrated interactions with antibiofilm molecules, suggesting its role in redox and biofilm modulation. This work aims to provide mechanistic insights into the antibiofilm mode of action of EA utilizing computational studies, biophysical measurements, enzyme inhibition studies on WrbA, and biofilm and reactive oxygen species assays exploiting a WrbA-deprived mutant strain of *Escherichia coli*. Our research efforts led us to propose that the antibiofilm mode of action of EA stems from its ability to perturb the bacterial redox homeostasis driven by WrbA. These findings shed new light on the antibiofilm properties of EA and could lead to the development of more effective treatments for biofilm-related infections.

## 1. Introduction

**Figure 1:**
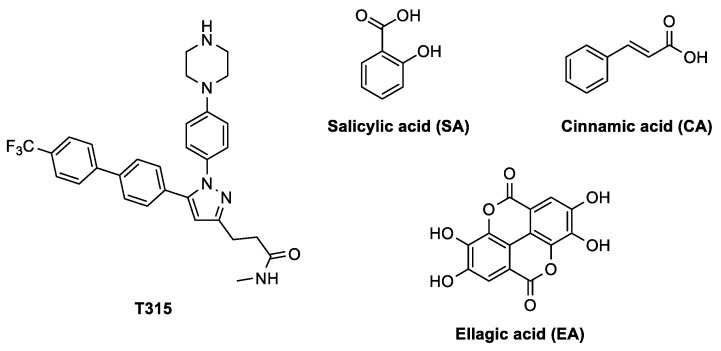
Chemical structure of known antibiofilm agents.

Biofilms are multicellular microbial communities embedded in an extracellular polymeric substances (EPS) matrix that can adhere to biotic or abiotic surfaces [1]. From an anthropocentric perspective, depending on the biofilm taxa and the colonized niches, biofilms can have either beneficial or detrimental effects [2]. In biofilms, microbial cells can tolerate stressful conditions such as low nutrient supply, oxidative environments, host defense mechanisms, or the presence of antimicrobial substances [3]. Because of their resilience, bacterial pathogen biofilms are a significant factor in the persistence of infections and are involved in most bacteria-caused diseases [3]. Hence, biofilm is an important target to be considered in addressing the fight against infections. Its impairment can strengthen the host’s defenses and the action of antibiotics, thus reducing the dose of administered antibiotics and the spread of antimicrobial resistance [4]. In this frame, the protein WrbA, tryptophan (W)-repressor binding protein A, appears to play a significant role in biofilm formation and modulation [5]. Furthermore, it has been identified as a potential biomarker for antibiofilm compounds [5]. This observation has been supported by functional validation studies that confirm the molecular targeting of WrbA by salicylic and cinnamic acids (hereinafter referred to as SA and CA, for brevity, Figure 1) in *Escherichia coli* [5]. Together with zosteric acid (ZA), these compounds have demonstrated antibiofilm activity in various studies [6,7,8,9]. WrbA was identified in *E. coli* as a protein interacting with SA and CA using a protein pull-down assay and mass spectrometry analyses. This finding was important in unraveling the molecular mechanism behind the antibiofilm properties of ZA [6,7], which is a natural *p*-sulphoxy-derivative of CA primarily found in marine plants such as eelgrass (e.g., *Zostera marina*) [8].

Functional genomic studies have revealed that the loss of WrbA in *E. coli* leads to a reduction in biofilm formation, which occurs through a ROS-dependent mechanism [5].

In planktonic *E. coli*, WrbA protein is upregulated by RpoS, the master transcriptional regulator of the stationary phase and the general stress response that was proven to also be involved in the architecture of biofilm [9]. Moreover, the WrbA protein is overexpressed under stressful conditions, such as mutant-induced overproduction of poly-β-hydroxybutyrate, in *Azotobacter vinelandii* [10]. In *E. coli*, the *wrb*A transcript is increased (i) in the presence of cold stress, a condition where expression of biofilm-related genes was also observed [11], (ii) in hyperosmotic and acidic pH stresses [12], and (iii) in the response induced by treatment with the antimicrobial 4,5-dihydroxy-2-cyclopenten-1-one [13]. Furthermore, it was shown that the promoter of the *wrb*A gene in *E. coli* contains at least one of the 20 CsgD-binding sites in the genome [14]. CsgD is the master regulator of biofilm formation and interferes with the formation and function of flagella, leading to the inhibition of planktonic growth and the switch to biofilm formation [14].

WrbA was identified as a stationary-phase protein in *E. coli* [15]. It was named based on the initial evidence of its copurification with the tryptophan repressor (TrpR) and considering the results of immunoprecipitation assays [15]. However, *E. coli* WrbA has no effect on DNA binding by TrpR [16]. Although a direct connection to TrpR was ruled out, an indirect link to tryptophan metabolism could derive from the involvement of indole, a well-known signaling molecule in the stationary phase [17] and in biofilm [18].

The *E. coli* WrbA (EC 1.6.5.2) is a multimeric flavin-mononucleotide (FMN)-containing flavodoxin-like protein [18], a founding member of the type IV group of NAD(P)H:quinone oxidoreductases (NQOs), which prefer NADH over NADPH as the electron donor substrate [19]. Being a type IV NQO, the *E. coli* WrbA is characterized by a unique αβ unit, contains one FMN molecule per monomer, and is in a dimer-tetramer equilibrium [19]. The presence of WrbA-like sequences in non-redundant databases of bacteria and fungi suggests the important role of these proteins in microbial life [6]. This role may be associated with the response against environmental stresses, including those related to host defense mechanisms in some cases of human infections or agricultural diseases [6]. Surprisingly, WrbA-like proteins are not conserved in mammalian cells [20]. WrbA from *E. coli*, as well as from *Archaeoglobus fulgidus* and other members of type IV NQOs, are thought to play a role in the oxidative stress protective response by preventing the interaction of the radical semiquinone with O_2_ and the subsequent production of ROS. Indeed, these NQOs can catalyze a two-electron reduction of quinone [19,21]. Functional studies showed that the NADH-dependent oxidoreductase function of purified *E. coli* WrbA is inhibited by the antibiofilm molecules SA and CA [5]. This finding is noteworthy as it experimentally supports the relationship between antibiofilm properties and stressful conditions, such as oxidative imbalance. This relationship was previously suggested by Villa and co-workers [22], who proposed that the antibiofilm molecule ZA acts as an environmental cue signaling global stress in *E. coli* cells.

Other natural compounds can hinder the bacteria’s ability to form and maintain biofilms, thereby overcoming antibiotic resistance associated with biofilm characteristics [23]. Together with these, the polyphenol ellagic acid (EA, Figure 1), reduces *E. coli* biofilm at 30–120 μg/mL [24,25]. Other studies investigated the antibiofilm properties of plant extracts containing EA. For example, the extract of pomegranate was shown to reduce *E. coli* and *C. albicans* biofilms [26]*,* while an extract of *Buchenavia tomentosa*, containing EA, displayed antibiofilm activity and low toxicity against *C. albicans* [27]. Nevertheless, the mechanism by which EA exerts its antibiofilm effect is still unknown. Ellagic acid (EA) is a naturally occurring bioactive polyphenolic compound. It is found as a secondary metabolite in numerous plant species, including pomegranate (*Punica granatum L*.), as well as in the wood and bark of certain tree species. EA is a dilactone derived from hexahydroxydiphenic acid (HHDP), which is a dimeric derivative of gallic acid. The hydrolysis of ellagitannins, a group of secondary metabolites found widely throughout nature, is the primary source of EA production. EA has gained significant attention for its notable anti-inflammatory, antimutagenic, and antiproliferative properties. It has demonstrated pharmacological effects in various in vitro and in vivo models [28].

Therefore, given the known antibiofilm property effect of EA and the established correlation between WrbA and biofilm, the primary objective of this study is to elucidate the mechanistic underpinnings of EA’s antibiofilm mode of action. The hypothesis is that EA exerts its effect by modulating the WrbA function. To verify this hypothesis, we have adopted a multidisciplinary approach including computational studies, biophysical measurements, evaluation of the impact of EA on WrbA enzyme function, and antibiofilm assays, along with reactive oxygen species (ROS) measurements.

## 2. Materials and Methods

### 2.1. Escherichia coli Strains and Growth Condition

The *Escherichia coli* strains BW25113 and BW25113-∆*wrbA* [5] were used as model systems for the bacterial biofilms. The strains were stored at −80 °C in suspensions containing 20% glycerol and 2% peptone and were routinely grown in Luria-Bertani broth (LB, Sigma-Aldrich, Saint Louis, MO, USA; Merck KGaA, Darmstadt, Germany) at 30 °C for 16 h.

### 2.2. Planktonic Growth in the Presence of EA

The planktonic growth of both wild-type and mutant *E. coli* strains was assessed in LB medium with 0 μM (negative control) and 500 μM of EA. EA (acquired from Molport, Riga, Latvia) stock in dimethyl sulfoxide (DMSO, Sigma-Aldrich, St. Louis, MO, USA) was used to obtain working solutions of EA with concentrations of 0.5 μM, 5 μM, 50 μM, or 500 μM; the starting stock was diluted in LB or other solutions based on the requirements of the assay. This was conducted in 96-well microtiter plates, and growth curves were generated at 37 °C using the Infinite^®^ F200 PRO microplate reader (TECAN, Mannedorf, Switzerland). Absorbance at 600 nm (A_600_) was measured every 10 min for over 24 h in wells containing 3 μL (3% *v*/*v*) of an overnight culture (final concentration 10^7^ cells/mL). To construct absorbance-based growth kinetics, the A_600_ of suspensions minus the A_600_ of the non-inoculated medium was plotted against the incubation time. The polynomial Gompertz model was used to fit the growth curves. The duration of the lag phase (λ, min), maximum specific growth rate (µ_m_, A_600_/min), and maximum growth (YM, A_600_) were calculated using XLSAT software (Version 2022.2.1, Addinsoft, Paris, France). Three biological replicates were performed for each treatment.

### 2.3. Adhesion Assay in the Presence of EA

A quantitative analysis was conducted to assess the effects of EA on cell adhesion using fluorochrome-labeled cells in hydrophobic 96-well black-sided plates, following the method previously reported by Villa et al. [29]. The microtiter plate wells were filled with 200 μL of phosphate-buffered saline (PBS, 0.01 M phosphate buffer, 0.0027 M potassium chloride pH 7.4; Sigma-Aldrich, Saint Louis, MO, USA) containing 10^7^ cells supplemented with 0 μM (negative control), 0.5 μM, 5 μM, 50 μM, or 500 μM of EA and incubated for 18 h at 37 °C. The wells were then washed twice with 200 μL of PBS and the adhered cells were stained with 10 µM SYTO™ 9 (ThermoFisher Scientific, Waltham, MA, USA) in PBS for 20 min in the dark at room temperature. Fluorescence intensity was measured using the Infinite^®^ F200 PRO microplate reader (TECAN) at an excitation wavelength of 483 nm and an emission wavelength of 503 nm. A standard curve of fluorescence intensity versus cell number was established and used to quantify the antibiofilm activity of EA. Six biological replicates of each condition were conducted. The biofilm inhibition performance was assessed by calculating the percentage reduction in the number of adhered cells relative to the control groups.

### 2.4. Level of Reactive Oxygen Species

The level of reactive oxygen species (ROS) experienced by both adhered and non-adhered cells with or without EA was quantified using the fluorogenic probe CellROX^®^ Green Reagent (ThermoFisher Scientific, Waltham, MA, USA). The cells were treated with CellROX^®^ reagent at a final concentration of 5 μM and incubated at room temperature for 30 min. Subsequently, the cells were washed twice with PBS and the fluorescence intensity was measured using the Infinite^®^ F200 PRO microplate reader (TECAN), with an absorption/emission maxima of 485/520 nm. To account for differences in cell numbers, the fluorescence intensity was normalized for both adhered and non-adhered cells. A total of six biological replicates were conducted for each condition to ensure statistical robustness.

### 2.5. Ligand Preparation, Grid Generation, and Docking Calculations

**Figure 2:**
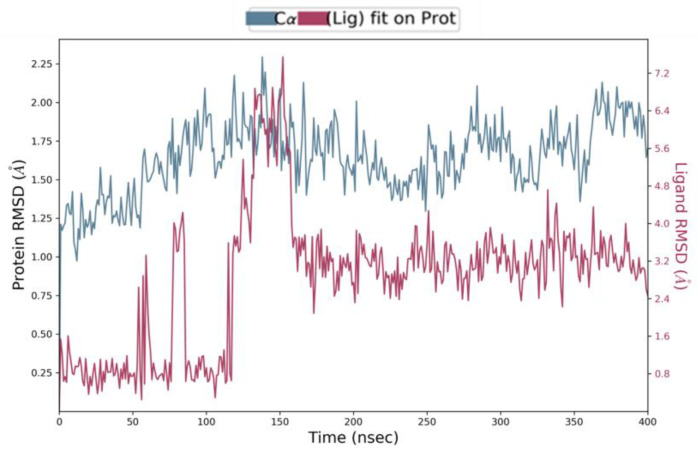
RMSD/time plot attained by MD simulations of WrbA/EA complex.

The structures of SA, CA (chosen as a reference compound), and EA were drawn by Maestro (Schrödinger LLC, New York, NY, USA) and then docked in the WrbA binding site, identified by the presence of benzoquinone in the X-ray crystal structure (PDB accession code 4YQE) [30]. The docking grid was centered between the co-factor FMN and Trp97, while the benzoquinone found in the X-ray was removed from the structure to allow the docking calculations of the ligands. The extra-precision (XP) docking protocol [31] was applied to achieve the most probable binding mode [32] of both compounds. Then, the WrbA complexes resulting from the best docking pose of each ligand were immersed in a box of water molecules, using the “system builder” tool of Maestro, and simulated by MD, using the Desmond algorithm. The MD simulation time was established considering the stability of the ligands in the catalytic site. To this aim, the Cα atom root mean square deviation (RMSD) *vs* time plots were considered (Figure 2). Then, 50 frames were extracted to calculate the ligand binding free energy values (ΔG) by Molecular Mechanics-Generalized Born Surface Area (MM-GBSA) [32,33]. In these calculations, the single trajectory approach was adopted, accepting the default parameters of the Prime Maestro protocol.

### 2.6. Purification of E. coli WrbA

The source for the purification of *E. coli* WrbA (WrbA_JW_) was the *E. coli* strain JW0989 ASKA (−) harboring the expression plasmid pCA24N*wrb*A(−) [34]. WrbA_JW_ contains a sequence identical to that of accession number P0A8G6 (*E. coli*, strain K12) equipped with an N-terminal His-tag. Overexpression of WrbA_JW_ and purification by ion metal affinity chromatography (IMAC) were carried out using published procedures [5]. Purified WrbA_JW_ in 50 mM NaH_2_PO_4_, 100 mM NaCl (pH 7.2) was stored ~2.4 mg/mL in small aliquots at −80 °C after a fast-freezing step with liquid nitrogen. Before use, an aliquot of protein preparation was gently thawed, and centrifuged (20 min, 4 °C, 13,000× *g*); soluble WrbA_JW_ was kept ~0.1 mg/mL at −20 °C in the presence of 20% glycerol for the month-term storage. The amount of protein was quantified by Bradford assay [35] using bovine serum albumin as the standard. The quality of overexpression and purification was checked using SDS-PAGE analyses. WrbA_JW_ functionality (1305 ± 99 U/mg) was verified by assaying the NADH:*p*-BQ oxidoreductase activity of the purified protein (0.05 µg/mL), following a published procedure [5], except for the modification of the final assay volume (500 µL) and for the presence of 50 µM FMN in the assay mixture.

### 2.7. Microscale Thermophoresis (MST)

The binding of EA to WrbA holoprotein (treated with an excess of FMN) was measured by MST [36]. WrbA_JW_ was labeled using a His-tag-specific dye (Monolith His-Tag Labeling Kit RED-tris-NTA 2nd Generation, MO-L018; NanoTemper Technologies GmbH, Munich, Germany) for 30 min at room temperature. FMN was then added to the labeled protein solution. The concentration of red-labeled WrbA and FMN was fixed at 35 nM and 70 nM, respectively, while one of the bindings partners (EA) ranged from 21.9 μM to 0.67 nM. MST analysis was assayed using the Monolith NT.115 instrument (NanoTemper Technologies GmbH, Munich, Germany) and applying the following experimental settings: Excitation Power of 80%, MST Power of 40% (medium), the temperature of 25 °C, and standard capillaries (consumables produced by NanoTemper Technologies GmbH, Munich, Germany). Both EA and WrbA/FMN complex were dissolved in PBS-T buffer (phosphate-buffered saline + 0.05% Tween™ 20) with 2.5% DMSO, and assayed after 15–40 min of incubation at room temperature. The auto-fluorescence of both FMN and EA was excluded before proceeding to the binding affinity assay. The dissociation constant (K_d_) was determined from compound concentration-dependent changes in normalized fluorescence (Fnorm), using the NanoTemper MO. Affinity Analysis software (version 2.3, NanoTemper Technologies GmbH, Munich, Germany) and applying the K_d_ model. Two independent experiments were carried out and the full analysis report is available in Figure S1 (see Supplementary Materials).

### 2.8. Surface Plasmon Resonance (SPR)

SPR assay was carried out using the BIAcore 8K system (Cytiva, Marlborough, MA, USA). The recombinant WrbA protein (3 μM) and the cofactor FMN (1 μM) were dissolved in 10 mM of sodium acetate (pH 5.5) and amine-coupled to a CM5 sensor chip (GE Healthcare, Chicago, IL, USA). The EA was dissolved in the running buffer (Sigma-Aldrich Dulbecco’s PBS, pH 7.4, 0.005% Tween™ and 5% DMSO; Sigma-Aldrich) at concentrations of 25 μM, 12.5 μM, 3.125 μM, 1.56 μM, 0.39 μM, and 0.19 μM, which were injected at a flow rate of 30 μL/min, with a contact time of 120 s, at 25 °C. The binding affinity was examined using the BIAevaluation software package (version 3.0.12, GE Healthcare), applying a steady-state model.

### 2.9. Inhibition Analysis of the WrbA Function

The inhibition of WrbA function was assayed by measuring the NADH:2,6-dichlorophenolindophenol (DCPIP) oxidoreductase activity of WrbA_JW_ in the presence of the potential inhibitor, using a published method [5] with some modifications. In these assays, reduced-nicotinamide adenine dinucleotide (NADH, N8129; Sigma-Aldrich, Saint Louis, MO, USA) is the electron donor substrate and the redox dye DCPIP (D1878; Sigma-Aldrich, Saint Louis, MO, USA) is the electron acceptor. The assays were carried out at room temperature in 96-well Greiner polystyrene microplates (M2936; Sigma-Aldrich, Saint Louis, MO, USA) by preparing an assay mixture (200 µL), whose components were added in sequence as follows: 0.5–0.6 µg/mL WrbA_JW_, assay buffer (50 mM Tris/HCl, pH 7.2), test molecule (0.0016–30 mM; 20–40 µL from stocks diluted in the proper diluent), 25 µM FMN (10 µL from 0.5 mM stock in assay buffer), 0.1 mM DCPIP (10 µL from very well-mixed 2 mM stock in assay buffer), and 0.2 mM NADH (10 µL from 4 mM stock in 50 mM Tris/HCl, 1 mM EDTA, pH 7.7). Molecules tested for inhibition were sodium salicylate (71945; Sigma-Aldrich, Saint Louis, MO, USA) prepared as 300 mM mother stock in assay buffer (final pH was 7.2), and EA prepared as 15 mM mother stock in polyethylene glycol 400 (PEG400 33124; Serva Feinbiochemica GmbH & Co., Heidelberg, Germany) well-dissolved by a 5-min treatment in an ultrasonic bath. Control reactions were set up in parallel either by replacing the test molecule with the proper stock diluent (assay buffer or PEG400) to make the not-inhibited enzyme reaction or by omitting WrbA_JW_ to exclude the non-enzymatic contribution from the measured reaction rates. After the addition of the last component (NADH), the microplate was mixed for 1 min in an orbital shaker (400 rpm), and rapidly placed in the microplate absorbance reader (iMark^™^, Bio-Rad, Hercules, CA, USA) to monitor the absorbance every minute for one hour using the 595 nm-filter. Initial rates of the absorbance decrease were calculated from quadratic regression fitted on absorbance values recorded in the first 15-min time window and were subtracted with corresponding non-enzymatic rates (see control above). The enzyme rate in the presence of the tested molecule (rate_molecule_) was compared to that achieved in the presence of the compound-diluent (rate_diluent_), in order to calculate the percent inhibition (I%) as follows:

I (%) = (rate_diluent_ − rate_molecule_/rate_diluent_) × 100 (1)

To quantify inhibition, four-parameter logistic curves were fitted through the SigmaPlot software (ver. 10; Systat Software Inc., San Jose, CA, USA) on data of measured activity plotted in function of different concentrations of the inhibitor, in order to extrapolate the inhibition concentration giving an activity half-way between minimum and maximum of the fitted inhibition curve (IC_50_).

### 2.10. Statistical Analysis

The analysis of variance (ANOVA) followed by Tukey’s honestly significant difference test (HSD) was conducted to determine significant differences among samples using the XLSAT software (Version 2022.2.1, Addinsoft). To establish whether a single group of data is significantly greater than the null value, a one-sample *t*-test was performed. Results were considered statistically significant if *p* values were equal to or less than 0.05.

## 3. Results

### 3.1. WrbA-Dependency of EA Antibiofilm Properties

**Figure 3:**
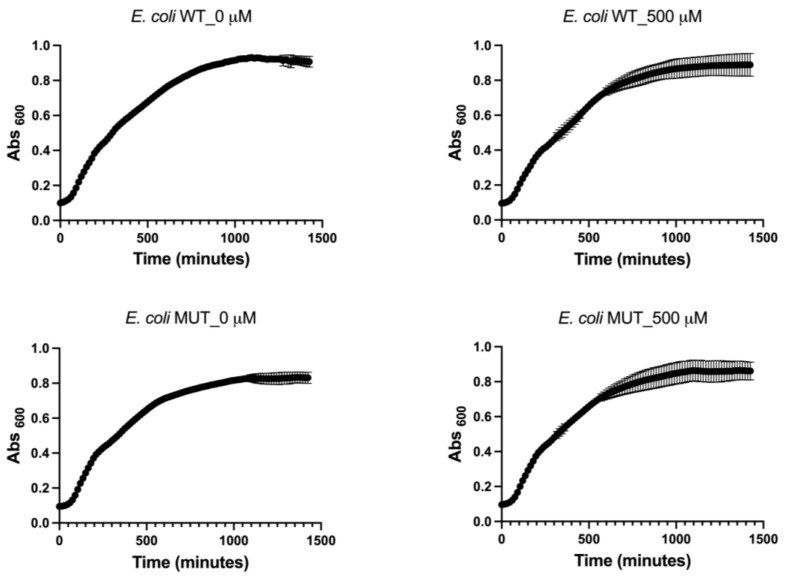
Planktonic growth of both wild-type (WT) and WrbA-deprived mutant (MUT) *E. coli* strains with and without the addition of 500 μM EA.

Prior to assessing the antibiofilm properties of EA, its capacity to function as a source of carbon and energy, as well as its potential effect on the planktonic growth of wild-type and WrbA-deprived *E. coli* strains, were examined. It was proved that EA could not serve as the sole carbon and energy source, as evidenced by the lack of growth observed in both bacterial strains when 500 μM EA was added to the mineral medium. To investigate the impact of EA on the planktonic growth of wild-type and mutant *E. coli* strains, we conducted experiments in a nutrient-rich medium with and without 500 μM EA. The growth curves and kinetic parameters obtained from these experiments are presented in Figure 3 and Table 1, respectively. The obtained results indicate that the maximum specific growth rate (µ_m_), and maximum growth (YM) remained unaltered in the presence or absence of the molecule (Table 1). Thus, the experiments revealed that EA, up to a concentration of 500 μM, did not affect *E. coli* planktonic growth.

**Figure 4:**
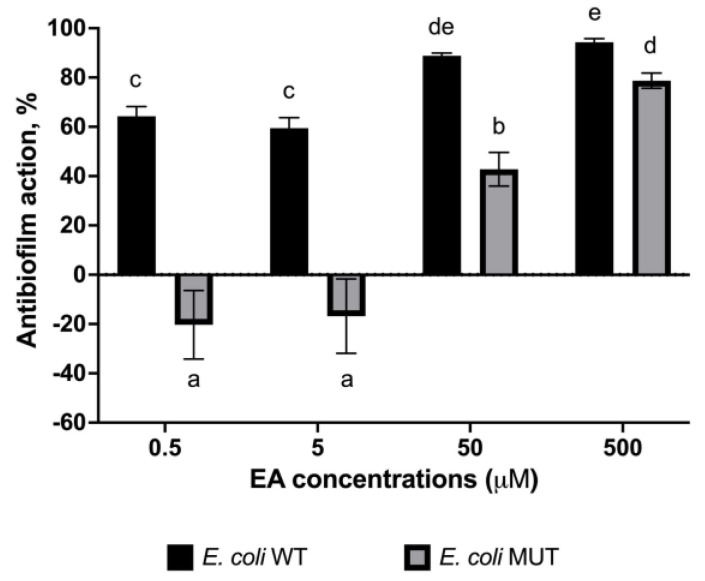
Antibiofilm effects of various concentrations of EA on both wild-type and WrbA-deprived mutant *E. coli* strains. To determine the antibiofilm effect, percentage values were calculated by comparing the treated samples to the corresponding control samples. The data represent the mean ± standard deviation of independent measurements. Statistically significant differences between conditions were determined using Tukey’s honestly significant difference test (HSD) with a significance level set at *p* ≤ 0.05. Different superscript letters were used to indicate significant differences between conditions.

The adhesion assay revealed that the antibiofilm efficacy of EA at non-lethal concentrations increases with the dosage, culminating in a maximum adhesion inhibition of 94% for the wild-type strain and 79% for the mutant strain at 500 μM EA (Figure 4). Of particular note is the finding that the antibiofilm efficacy of EA decreased in the WrbA-deprived mutant, with the most pronounced effect seen at the lowest EA concentrations, at which the molecule promoted cell adhesion by 19% and 16% at 0.5 μM and 5 μM EA, respectively (Figure 4). Thus, the significant decrease in the antibiofilm efficacy of EA was due to the absence of WrbA. In the WrbA-deprived mutant strain, EA influenced cell adhesion only at ≥50 µM concentrations, albeit to a lesser extent compared to the wild-type strain.

**Figure 5:**
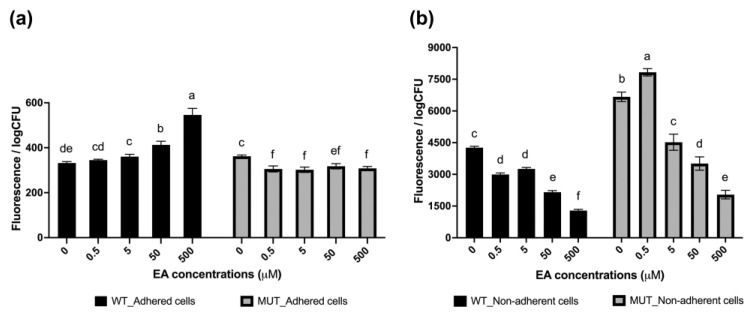
Levels of ROS experienced by the adhered cells (**a**) and non-adherent cells (**b**) of both wild-type and WrbA-deprived mutant *E. coli* strains exposed to non-lethal concentrations of EA. The data represent the mean ± standard deviation of independent measurements. Statistically significant differences between conditions were determined using Tukey’s honestly significant difference test (HSD) with a significance level set at *p* ≤ 0.05. Different superscript letters were used to indicate significant differences between conditions.

The ROS level experienced by both adhered (biofilm-forming) and non-adherent (planktonic) cells in the presence or absence of EA was also investigated (Figure 5). The results demonstrated that adhered cells of both *E. coli* strains exhibited low levels of ROS, whereas non-adherent cells experienced high levels of ROS. However, these levels decreased with increasing concentrations of EA. Notably, the mutant non-adherent cells exhibited 1.5–2 times higher levels of ROS than the wild-type strain.

### 3.2. In Silico Studies of EA

**Figure 6:**
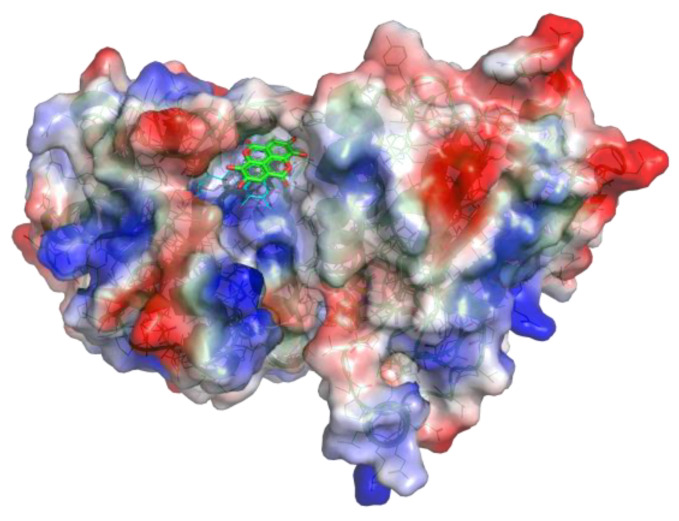
WrbA/EA complex resulting from the docking and MD simulations. The solvent-accessible surface of WrbA is colored depending on the partial charge of the atoms: the positive areas are depicted as blue, while the red areas suggest the presence of negatively charged residues. The carbon atoms of EA are represented as green sticks, and the ones of FMN are represented as cyan lines.

Computational studies were performed to ascertain if EA could bind to WrbA by establishing stable interactions over molecular dynamics (MD) simulations. Firstly, docking calculations permitted us to hypothesize the binding mode of EA to WrbA and to create the complex needed for the MD simulations. In particular, the WrbA/EA complex, obtained with the best EA docking solution (Figure 6), was solvated by water molecules, also adding the counter ions required to neutralize the overall charge of the simulating systems. Finally, 400 ns-long MD simulations were carried out, also evaluating the stability of the ligand by the RMSD/time plot in the active site of the enzyme (Figure 2). The MM-GBSA approach was then applied to estimate the ligand binding free energy value, performing calculations on the 50 MD frames in which EA displayed the highest stability, providing a ΔG value of −70.3 kcal/mol (SD = 4.7).

To compare this value to those of the reference compounds, SA and CA they were docked in the WrbA catalytic site, and the resulting complexes were simulated by MD, adopting the computational protocol previously described for EA. SA lost its interaction with the target within the initial 50 ns of MD simulations, while CA remained anchored in the catalytic site, displaying an estimated ΔG value of −31.5 kcal/mol (SD = 2.3). As can be noted, EA exhibited a predicted ΔG value significantly lower than that of CA, providing the first evidence that it can putatively interact with the WrbA catalytic site.

**Figure 7:**
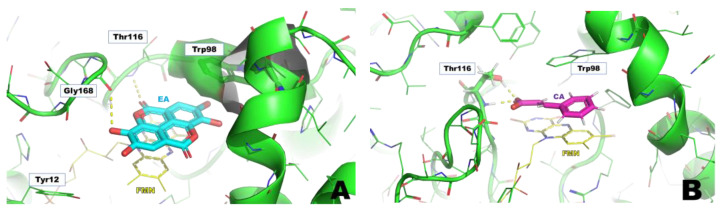
Supposed binding modes of EA (**A**) and CA (**B**) in complex with WrbA. Residues were numbered accordingly with the sequence reported on the Uniprot database (https://www.uniprot.org (accessed on 23 June 2022)), entry code P0A8G6.

After the MD simulations, a comparison of the binding modes (Figure 7) revealed that EA established sandwich π-π interactions with the tricyclic core of the FMN and the side chain of Trp98, mimicking the binding mode of benzoquinone observed in X-ray studies. Moreover, a hydrogen bond network was shaped by the hydroxyl and carbonyl groups of EA and the backbone atoms of Thr116, Gly168, and (in many MD frames) the side chain of Tyr12 (Figure 7A). Furthermore, the oxygen atoms of EA created numerous water-bridged H-bonds with WrbA (Figure 7A). Conversely, considering the simulations on the reference compound CA, the presence of a single aromatic ring prevented the formation of strong π–π interactions with FMN and Trp98. Moreover, the carboxylic group of CA established only two H-bonds with Thr116 (Figure 7B).

### 3.3. EA Binds to WrbA Protein, Biophysical Assays

**Figure 8:**
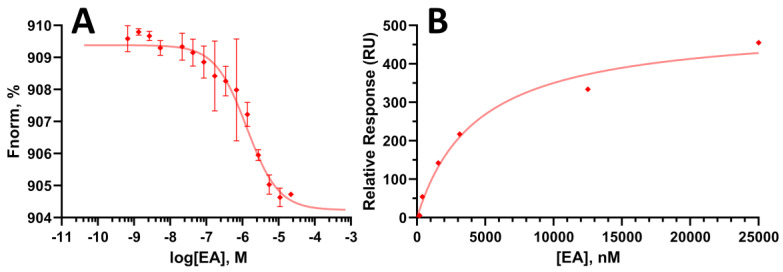
Binding affinity assays of EA on WrbA_JW_. (**A**) MST analysis and (**B**) SPR curve of the interaction between EA and WrbA_JW_ (K_d_ = 1.5 ± 0.4 μM and 4.5 μM, respectively). Both figures were generated using GraphPad Prism software (version 8.0.2, GraphPad Software Inc., San Diego, CA, USA).

Biophysical experiments were conducted by MST and SPR to demonstrate the ability of EA to bind WrbA and to measure the binding affinity expressed as K_d_ value (see Material and Methods section for details). In the MST tests, EA exhibited a K_d_ value of 1.5 ± 0.4 μM (Figure 8A), while the SPR measurements revealed a K_d_ value of 4.5 μM (Figure 8B). As can be noted, the high value of RU could indicate a non-specific binding of EA but, to exclude this possibility, we analyzed the sensorgram resulting from the injection of EA in both flow cells of the CM5 chip, the first containing the immobilized WrbA and the other the carboxylmethylated dextran matrix, see Figure S2 (see Supporting Material). The attained results clearly indicate that there is a specific binding in the protein-containing cell, since the signal remained constant in the presence of increasing EA concentrations (Figure S2A, Supporting Material). Considering that, upon completion of the immobilization process, we obtained a final Response Units (RU) value of 7900 and consequently the RUmax attributable to the ligand falling within the range of 100 to 150 RU. Consequently, these findings suggest a ligand-protein binding stoichiometry of 1:1 at the Kd concentration range of EA. Conversely, at high concentrations, the ligand-protein stoichiometry can reach 3:1, maybe due to the planar structure of EA, that could lead to multiple stacking on the FMN present in the catalytic site of WrbA.

### 3.4. WrbA Function Is Inhibited by EA

**Figure 9:**
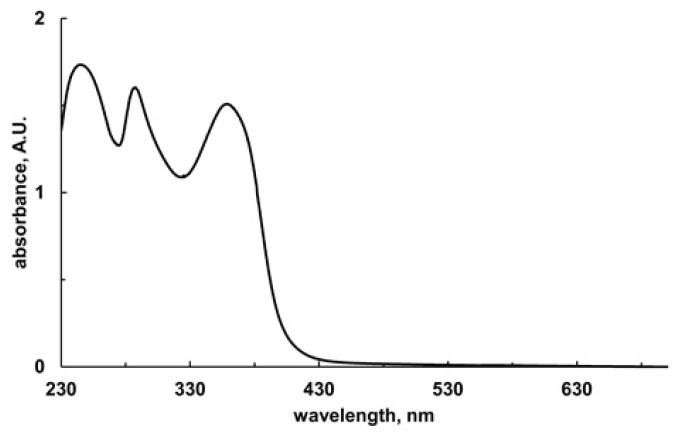
The UV/visible spectrum of EA. The spectrum was collected in a 1-cm quartz cuvette containing 0.8 µM EA in 50 mM Tris/HCl (pH 7.2), 20% PEG400. Absorbance values measured at 340 and 595 nm were 1.267 and 0.008, respectively. Absorbance maxima of EA were found to be at the 248, 287, and 359 nm wavelengths.

In previous work, it was shown that SA (4 mM) inhibits the NADH:*p*-benzoquinone oxidoreductase activity of the *E. coli* recombinant form WrbA_JW_ [5]. Here, the NADH-dependent oxidoreductase function of WrbA_JW_ was measured using a spectrophotometric method, based on the redox dye 2,6-dichlorophenolindophenol (DCPIP) as an electron acceptor. This method was used to extend the enzyme inhibition analysis to tested molecules with strong absorbance in the near UV wavelength range. EA showed a very high absorbance at 340 nm (ε_340_ = 1584 mM^−1^ cm^−1^), whereas a much lower absorbance was measured at 595 nm (ε_595_ = 10 mM^−1^ cm^−1^) (Figure 9). The latter was the wavelength used in the assay because it is associated to DCPIP reduction.

**Figure 10:**
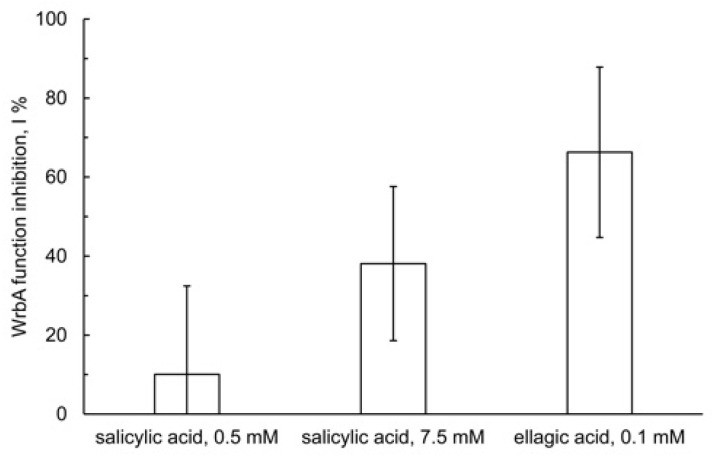
Enzyme inhibition of the WrbA function. The NADH:2,6-dichlorophenolindophenol oxidoreductase activity was detected at 25 °C in assay buffer (50 mM Tris/HCl, pH 7.2), containing purified *E. coli* WrbA_JW_ (0.5 µg/mL), tested molecules as indicated, 25 µM FMN, 0.1 mM 2,6-dichlorophenolindophenol, and 0.2 mM NADH. The value of the change in activity due to the tested molecule was percent-related to the uninhibited activity (I %) of a control reaction carried out in the absence of the tested molecule (replaced by the stock diluent: assay buffer, in the case of SA; PEG400, in the case of EA); data are represented as means with standard deviations.

As reported in Figure 10, the NADH:DCPIP oxidoreductase activity of WrbA_JW_ was found to be inhibited by SA. The inhibition is evident in the presence of 7.5 mM SA with an inhibition percent (I%) value of 38.1 ± 19.5 % (*p* = 0.0013), whereas it was not detectable at 0.5 mM SA (*p* = 0.2718). In these measurements, EA was tested at 0.1 mM, and inhibition of NADH:DCPIP oxidoreductase activity of WrbA_JW_ was clearly detectable with an I% value of 66.3 ± 21.6 % (*p* < 0.0001; Figure 10).

To quantify the inhibition of the NADH:DCPIP oxidoreductase function of WrbA_JW_, the activity was determined in the presence of different concentrations of the tested molecules. Replicates of inhibition curves were used to generate IC_50_ and MIC values. The IC_50_ value of EA was 22 ± 15 µM, 357-fold lower than that detected for salicylic acid (7.85 ± 2.65 mM) (*p* < 0.0001) (Table 2). The MIC value of EA was 382-fold lower than that of SA (11 ± 9 µM vs. 4.20 ± 1.84 mM) (*p* = 0.0007). These data provide clear evidence that EA acts as a much stronger inhibitor of the WrbA function than SA.

## 4. Discussion

This study showed that even at the lowest tested concentration of 0.5 µM, EA caused a significant 2.8-fold decrease in *E. coli* adhesion. Importantly, this concentration was found to be sub-lethal, as it did not cause any impairment of the *E. coli* planktonic growth (>500 µM, this work; >3300 µM, [24]; >2000 µM, [25]; >260 µM, [30]), indicating that the observed effect on adhesion was specific and not due to general growth inhibition. Therefore, our findings confirm the antibiofilm properties of EA, consistent with previous studies conducted on clinical *E. coli* strains at concentrations of 100 µM [25] or 400 µM [24], as well as in *E. coli* ATCC 10536 at a concentration of 17 µM [30]. In our experiments, EA led to a decrease in *E. coli* biofilm by 60–94% in the concentration range of 0.5–500 µM. Notably, at a concentration of 0.5 µM, EA exhibited a similar antibiofilm effect to that of SA at a concentration approximately 370-fold higher (183 µM, *E. coli* K-12 wild-type ATCC25404 strain; [7]).

The heterologous expression of *E. coli* WrbA in *Candida albicans* restored the sensitivity to oxidative agents (e.g., H_2_O_2_, menadione) in mutant strains lacking the four *C. albicans wrb*A-like genes. This result demonstrated that WrbA-like proteins play a key antioxidant role in *C. albicans* as well [37]. Furthermore, in a mouse model of hematogenously disseminated candidiasis, the virulence-promoting effect of *C. albicans wrb*A-like genes was demonstrated [37]. Another study revealed the involvement of WrbA in the antibiofilm effect of T315 (Figure 1), a phenyl-pyrazole analog, in *Salmonella enterica* serovar Typhi, the etiological agent of typhoid fever, which relies on biofilm formation for its persistence [38]. The importance of WrbA in the pathogenesis of the Shiga toxin-producing foodborne pathogen *E. coli* O157:H7 is confirmed by the identification of its gene as a target for DNA insertions or deletions, contributing to the diversity of clinical strains [39]. Additionally, WrbA has been found to be the target of salicylidene acylhydrazides, antivirulence drugs that affect the Type III Secretion System and to enable colonization of the host gastrointestinal tract by the enterohaemorrhagic *E. coli* pathotype [40]. WrbA has also been implicated in the pathogenesis of *Pseudomonas aeruginosa* in mammals [41]. Considering the findings in *E. coli* [5], it is possible that the involvement of WrbA in the three last aforementioned examples is related to biofilm formation, a crucial step for host colonization. Taking into account the evidence of WrbA’s role in biofilm formation, along with the known antibiofilm properties of molecules that interact with WrbA and inhibit its function [5,7], we hypothesized that WrbA could serve as a potential target for the antibiofilm ability of EA.

The present study demonstrated that the antibiofilm effect of EA was substantially reduced in the WrbA-deprived *E. coli* mutant strain (BW25113-∆*wrb*A) compared to the wild-type strain. Notably, this effect was particularly pronounced at lower concentrations, at which exposure to 5 or 0.5 µM EA resulted in a significant increase in the number of attached cells lacking the WrbA protein (pro-biofilm effect). These data have shown that the antibiofilm effect of EA is highly dependent on the presence of WrbA, especially at concentrations of 5 µM or lower. However, at higher concentrations (≥50 µM), the dependence of EA on WrbA appeared to be reduced, implying the involvement, at these concentrations, of additional mechanisms, which could not rely on catalytic partners or effectors, such as the WrbA enzyme.

The structural models built on the X-ray 3D structure of *E. coli* WrbA led us to suppose that EA could bind to WrbA. Our docking calculations revealed that EA possessed a higher docking score and a lower predicted binding free energy value compared to CA, used as a reference. In particular, the predicted ΔG value of EA was almost 40 kcal/mol lower than that of CA.

Biophysical studies, which involved SPR and MST experiments, corroborated the computational outcomes since confirmed that EA can bind on WrbA. In detail, the measurement of the K_d_ of EA revealed values in the low micromolar range (1.5–4.5 µM). Moreover, enzyme inhibition studies carried out in this work further confirmed that EA inhibits the NADH-dependent oxidoreductase function of WrbA. The observed inhibition was characterized by a MIC value in the micromolar range. The inhibitory effect of EA was ~380-fold higher than that of SA, in line with their anti-biofilm properties. Our findings provided compelling evidence of the interaction between EA and WrbA and its impact on the NADH-dependent oxidoreductase function of WrbA. Previously, Moshiri et al. [38] demonstrated that the action of T315 on *S. enterica* biofilm was dependent on WrbA by using a WrbA-deprived mutant strain. However, the cause of this dependency was not elucidated. In another study, Rossi et al. [5] showed for the first time that the antibiofilm molecules SA and CA affect the WrbA function. However, a WrbA-dependency of their antibiofilm properties was not proved. Our study provides the first evidence that the antibiofilm effect of EA, a molecule of increasing pharmaceutical interest, primarily relies on WrbA and is linked to the inhibition of WrbA enzyme function resulting from the interaction between EA and WrbA.

Based on these findings, two possible mechanistic models for the antibiofilm mode of action of EA can be proposed. The first model considers WrbA as a critical “biofilm factor” that maintains redox homeostasis, especially during biofilm formation. In this model, the inhibition of WrbA by EA leads to an imbalance in redox homeostasis, compromising the biofilm’s ability to maintain a stress-protective environment. Alternatively, WrbA could act as a “redox-alerting probe”; this second, more complex model assumes WrbA role in redox alerting. In this case, the interaction between EA and WrbA would lead to a redox alert, elevating the basal antioxidant activity in planktonic cells and making the stress-protective biofilm lifestyle unnecessary.

The non-adherent (planktonic) cells of *E. coli* wild-type strain had lower ROS levels upon exposition to EA at submicromolar concentrations. On the other hand, this effect was not observable in planktonic cells of the WrbA-deprived mutant strain, where ROS levels were higher in the presence of EA at submicromolar concentrations compared to the control (which was not treated with EA). Consistently with a “redox-alerting probe” role of WrbA, these findings demonstrate that the inhibition of WrbA by EA at submicromolar concentrations triggers a significant cellular antioxidant response that allows cells to maintain redox homeostasis, without relying on the protective environment of the biofilm lifestyle. Consequently, under these conditions, the wild-type strain exhibited a significant decrease of approximately 1.7-fold in biofilm formation. On the other hand, in WrbA-deprived planktonic cells, the presence of submicromolar EA led to an oxidative imbalance, which forced the cells to seek refuge in the biofilm lifestyle. This would explain the observed pro-biofilm effect of EA in the absence of WrbA.

In the case of adhered cells (both wild-type and mutant cells), the ROS levels were at least 10-fold lower compared to those experienced by the planktonic cells. Regarding the adhered cells of the wild-type *E. coli* strain, we observed a statistically significant increase in ROS levels at concentrations of 50 µM or higher. Consistently with a “biofilm factor” role of WrbA, our findings suggest that at high concentrations of EA, WrbA function in maintaining redox homeostasis is considerably inhibited. Conversely, in the attached cells of the mutant *E. coli* strain lacking WrbA, the ROS levels remained mostly unchanged across all EA-tested concentrations. In this case, it is plausible that EA cannot interfere with the antioxidant function of WrbA, which is absent in the mutant strain, and compensatory antioxidant mechanisms are activated instead. These findings underscore the complexity of the antibiofilm mechanism of EA and the intricate interplay between WrbA and oxidative imbalance.

Overall, our analysis of the effects of EA on biofilm formation, its interaction with WrbA, and the differences in response among the tested *E. coli* strains, along with the observed inhibition of WrbA function by EA, led us to propose two possible explanations for the antibiofilm mode of action of EA based on ROS levels. First, the inhibition of WrbA function may lead to an elevated generation of ROS, which then triggers antioxidant mechanisms in planktonic cells, rendering the protective biofilm lifestyle useless. Second, WrbA may play a crucial role in the redox homeostasis of biofilm, and its inhibition by EA may make the biofilm lifestyle unsustainable. The first explanation is supported by ROS data in planktonic cells at submicromolar EA, whereas the second explanation is indicated by ROS data in biofilm at high EA concentrations.

The WrbA-based mechanism of the antibiofilm properties of EA, not only provides a possible explanation for the observed antibiofilm effects in studies using pomegranate peel extract [30] or *Bauhinia tomentosa* [31], where EA is a major component but also sheds light on the mode of action of other antibiofilm compounds. For instance, the WrbA-dependent antibiofilm action of T315 against *S. enterica* biofilm was reported by Moshiri et al. [38], and the effect of WrbA-interacting antibiofilm compounds, like SA, CA [5] as well as ZA, caffeic acid, and coumaric acid [6], believed to interact with WrbA, could potentially be attributed to the same mechanism of action.

## 5. Conclusions

In this work, the interaction of EA with the NADH:quinone oxidoreductase WrbA was demonstrated both in silico and through biophysical analyses. Furthermore, it was found that this interaction causes the inhibition of the enzyme function of WrbA. Taking advantage of the WrbA-deprived mutant *E. coli* strain, we demonstrated that both the antibiofilm properties of EA and the observed variations in ROS levels are dependent on WrbA. These findings led us to propose that the antibiofilm mode of action of EA is a result of the perturbation of WrbA-driven redox homeostasis. The mechanistic insights gained from this research provide a solid foundation for future studies exploring the potential use of EA in novel non-biocidal strategies against human pathogens, which decreases the selective pressure contributing to antibiotic resistance. Such strategies hold promise in mitigating the rise of drug-resistant bacteria, thereby offering effective and sustainable solutions to address the growing problem of antibiotic resistance.

## Figures and Tables

**Table 1:** The maximum specific growth rate (μ_m_), maximum growth (YM), and goodness of fit (R^2^) were determined from the planktonic growth curves of both wild-type and WrbA-deprived mutant *E. coli* strains. The Gompertz model was employed to calculate these growth parameters, considering the presence or absence of 500 μM EA. The data presented in the table are the mean values ± SDs of independent measurements. No significant differences between the means were found (*p* > 0.05).

	WT 0 µM EA	WT 500 µM EA	MUT 0 µM EA	MUT 500 µM EA	*p*-Value
µ_m_	1.42 × 10^−3^ ± 1.83 × 10^−7^	1.42 × 10^−3^ ± 3.46 × 10^−6^	1.42 × 10^−3^ ± 3.42 × 10^−6^	1.42 × 10^−3^ ± 9.84 × 10^−7^	0.518
YM	0.938 ± 0.013	0.901 ± 0.064	0.834 ± 0.033	0.869 ± 0.067	0.143
R^2^	0.9945	0.9792	0.9920	0.9745	

**Table 2:** Quantification parameters of WrbA_JW_ enzyme inhibition.

Compound	IC_50_, mM ^a^	MIC, mM ^b^
SA ^c^	7.848 ± 2.650	4.199 ± 1.839
EA	0.022 ± 0.015	0.011 ± 0.009

^a^ IC_50_, the concentration giving the half-way response between the minimum and maximum of the inhibition curve fitted on experimental data of the NADH:DCPIP oxidoreductase activity of 0.5 µg/mL WrbA_JW_ in the presence of different concentrations of the tested compounds. Reported values are the mean ± SD of seven replicated inhibition curves. ^b^ MIC, the minimal inhibition concentration giving an appreciable decrease of the measured enzyme activity (i.e., the maximum activity value from the curve minus the SD of the control, where tested compounds were omitted). ^c^ SA is delivered as sodium salicylate; reported values are the mean ± SD of at least three replicated inhibition curves.

## Data Availability

Not applicable.

## Supplementary Materials

The following supporting information can be downloaded at: https://www.mdpi.com/article/10.3390/pharmaceutics15061757/s1, Figure S1: MST binding affinity assay report of EA on WrbA_JW_; Figure S2: (A) Original sensorgram for the SPR analysis carried out to investigate the binding of the EA to SPR. (B) Original reference subtracted sensorgram for the SPR analysis carried out to investigate the binding of the EA.
