# Supplementary material for: Mechanistic Insights into the Antibiofilm Mode of Action of Ellagic Acid
Source: Pharmaceutics. 2023 Jun 17;15(6):1757. doi: 10.3390/pharmaceutics15061757 (PMC10302398; doi:10.3390/pharmaceutics15061757)
Supplement: Supplementary file 1 [file pharmaceutics-15-01757-s001.zip › pharmaceutics-2419511-supplementary.pdf]

Supporting Material

for

Mechanistic Insights into the Antibiofilm Mode of Action of Ellagic Acid

Alessandro Ratti, Enrico M. A. Fassi, Fabio Forlani, Matteo Mori, Federica Villa, Francesca Cappitelli, Jacopo Sgrignani, Gabriella Roda, Andrea Cavalli, Stefania Villa, and Giovanni Grazioso

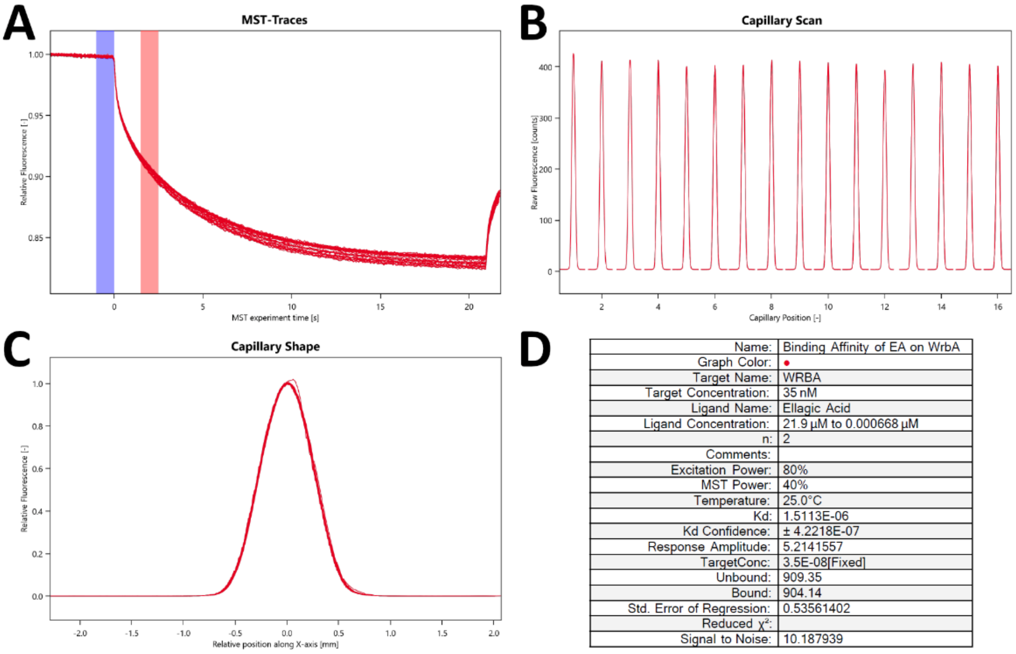

Name:	Binding Affinity of EA on WrbA
Graph Color:	•
Target Name:	WRBA
Target Concentration:	35 nM
Ligand Name:	Ellagic Acid
Ligand Concentration:	21.9 µM to 0.000668 µM
n:	2
Comments:	
Excitation Power:	80%
MST Power:	40%
Temperature:	25.0°C
Kd:	1.5113E-06
Kd Confidence:	± 4.2218E-07
Response Amplitude:	5.2141557
TargetConc:	3.5E-08[Fixed]
Unbound:	909.35
Bound:	904.14
Std. Error of Regression:	0.53561402
Reduced χ^2:	
Signal to Noise:	10.187939

Figure S1. MST binding affinity assay report of EA on $WrbA_{JW}$. (A) MST traces, (B) raw fluorescence values, and (C) capillary shape displayed by each capillary. (D) Dataset overview.

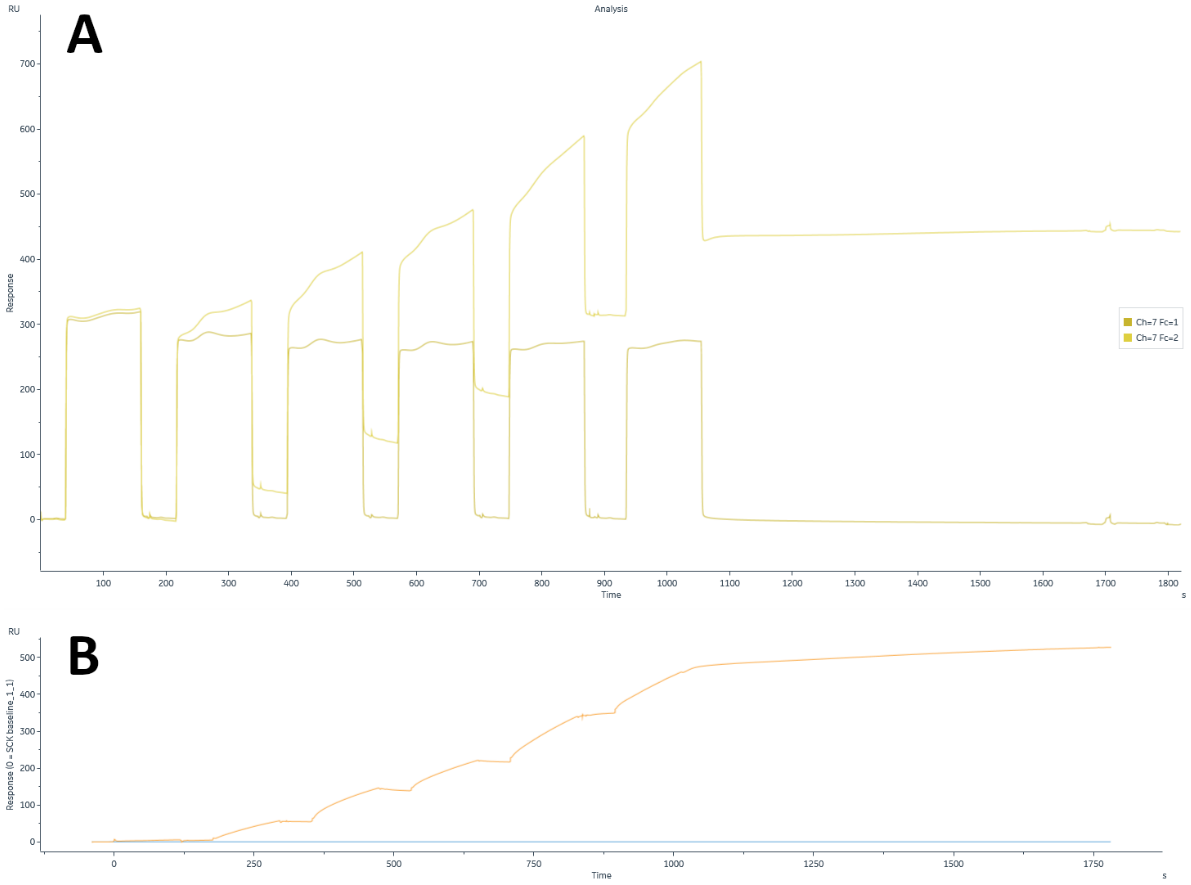

Figure S2. (**A**) Original sensorgram for the SPR analysis carried out to investigate the binding of the EA to SPR. The Flow cell (FC1) is the reference, while FC2 contains the immobilized WrbA. (**B**) Original reference subtracted sensorgram for the SPR analysis carried out to investigate the binding of the EA. The orange line corresponds to the EA injection, while the blue one is the running buffer injection (Blank).
